# The therapeutic efficacy of ^225^Ac-DOTATATE in neuroendocrine tumors: a preliminary meta-analysis

**DOI:** 10.3389/fonc.2025.1696063

**Published:** 2025-10-31

**Authors:** Jiao Ma, Yang Ji, Zhihan Yao, Jiangchu Yangqing, Chunyin Zhang

**Affiliations:** ^1^ Department of Nuclear Medicine, Affiliated Hospital of Southwest Medical University, Luzhou, Sichuan, China; ^2^ Nuclear Medicine and Theranostics Key Laboratory of Sichuan Province, Luzhou, Sichuan, China; ^3^ Institute of Nuclear Medicine, Southwest Medical University, Luzhou, Sichuan, China

**Keywords:** neuroendocrine tumors, ^225^Ac-DOTATATE, efficacy, meta-analysis, radionuclide therapy

## Abstract

**Objective:**

This meta-analysis aims to evaluate the therapeutic efficacy and toxicity of ^225^Ac-DOTATATE in patients with metastatic neuroendocrine tumors (NETs).

**Methods:**

This systematic review adheres to the Preferred Reporting Items for Systematic Reviews and Meta-Analysis (PRISMA) guidelines. PubMed and Embase were searched to identify studies that met the inclusion criteria. The primary endpoints were the evaluation of therapeutic efficacy through disease response rates (DRRs) and disease control rates (DCRs), and then toxicity is assessed. Additionally, a subgroup analysis was performed to evaluate the influence of prior ^177^Lu-peptide receptor radionuclide therapy (PRRT) on efficacy.

**Results:**

This meta-analysis included five studies involving a total of 153 patients. The results showed that the DRR following ^225^Ac-DOTATATE treatment was 52% [95% confidence interval (CI): 43%–61%], and the DCR was 88% (95% CI: 81%–94%). The incidence of hematological toxicity was low at 2% (95% CI: 0.00%–5%), with only two patients experiencing Grade I–II renal toxicity, and no Grade III–IV toxicities were observed. Subgroup analysis indicated that patients who had previously received ^177^Lu-PRRT treatment had a DRR of 51% (95% CI: 35%–66%) and a DCR of 90% (95% CI: 69%–100%), while ^177^Lu-naive patients had a DRR of 47% (95% CI: 1%–97%) and a DCR of 89% (95% CI: 72%–100%).

**Conclusion:**

Our preliminary analysis shows that ^225^Ac-DOTATATE is an effective and safe treatment option for advanced metastatic NETs, significantly improving patients’ quality of life and demonstrating considerable disease control even in cases where other treatments have failed.

**Systematic review registration:**

https://www.crd.york.ac.uk/prospero/, identifier CRD42025633806.

## Introduction

Neuroendocrine tumors (NETs) are a heterogeneous group of neoplasms originating from neuroendocrine cells, commonly occurring in the gastrointestinal tract, pancreas, and stomach. The incidence of NETs has been steadily increasing in recent years (1, 2). Traditional therapeutic approaches mainly include surgery, endocrine therapy, targeted chemotherapeutic agents, and radiochemotherapy (3). Despite recent progress in the diagnosis and treatment of NETs, therapeutic options remain limited for patients with advanced or metastatic disease, and their prognosis is generally poor, highlighting an urgent need for new treatment strategies (4).

Emerging peptide receptor radionuclide therapy (PRRT) has garnered significant research attention due to its demonstrated efficacy in NET treatment, with radiolabeled somatostatin analogs (e.g., DOTATATE) being the most widely applied (5, 6). The β-emitting radionuclide ^177^Lu is currently the most commonly used, with ^177^Lu-DOTATATE receiving Food and Drug Administration (FDA) approval in 2018 for the treatment of metastatic NETs (5). However, studies have shown that even patients with high somatostatin receptor expression and an initially favorable response to ^177^Lu-DOTATATE eventually develop resistance to this β-emitting PRRT, resulting in disease progression (7, 8).

Targeted alpha therapy (TAT) has emerged as a promising alternative to β-emitting radionuclides, with ^225^Ac being the most widely studied alpha-emitting radionuclide (9, 10). Compared to ^177^Lu, ^225^Ac (*T*_1/2_ = 9.9 days), as a high-energy (5.8–8.4 MeV) and short-range (47–85 μm) alpha emitter, exhibits significantly higher linear energy transfer (LET ≈ 100 keV/μm), allowing for potent tumoricidal effects with relatively minimal damage to surrounding normal tissues (10–12). Preliminary studies suggest that ^225^Ac-DOTATATE offers superior potential in targeting NETs, making it a promising alternative to ^177^Lu-based therapies (13, 14).

However, clinical studies on ^225^Ac-DOTATATE for NETs remain limited, with small sample sizes and inconsistent findings. Thus, this meta-analysis aims to systematically evaluate the safety and efficacy of ^225^Ac-DOTATATE in the treatment of NETs, providing robust evidence for clinical practice and a reference for future TAT research.

## Materials and methods

This systematic review followed the Preferred Reporting Items for Systematic Reviews and Meta-Analysis (PRISMA) guidelines (15). The registration number on the International Prospective Register of Systematic Reviews (PROSPERO) is CRD42025633806.

### Search strategy

A systematic search was conducted in PubMed and Embase from establishment to 15 December 2024. The search terms were as follows: “^225^Ac-DOTATATAE” AND (“neuroendocrine tumor” [Mesh] OR “neuroendocrine tumour*” OR “neuroendocrine neoplasm*” OR “neuroendocrine cancer*” OR “neuroendocrine carcinoma*”). Two researchers independently screened the literature and extracted data. Eventually, they selected the studies to be finally included and the data extraction results through a unanimous agreement. In case of disagreement, a third party is consulted in order to reach a consensus.

### Study selection and quality assessment

The search was limited to human studies published in English. The studies discussed the treatment efficacy and toxicity of ^225^Ac-DOTATATE that meet the following criteria: (1) Patients confirmed neuroendocrine tumors by biopsy, laboratory examination, and imaging examination; (2) patients with incomplete or unresectable tumors, postoperative tumor recurrence, and distant metastases, as well as patients who were either treatment-naive or resistant to conventional therapies or ^177^Lu-PRRT were included; and (3) baseline ^68^Ga-DOTATATE/DOTANOC PET/CT scan showed high somatostatin receptor (SSTR) expression (uptake greater than the liver). Studies about animal experiments, cell studies, reviews, meta-analyses, replications, case reports, or letters were excluded. The quality of these studies was assessed based on the JBI Critical Appraisal Checklist for Case Series (16).

### Data extraction

The data extracted from the chosen studies included the following: basic characteristics (the first author, publication time, treatment response criteria, number of patients, gender, type of primary tumor, Ki-67 index, previous treatment methods, and metastatic site), treatment details (dose, total cycles, interval time, follow-up time, and cumulative activity), and therapeutic efficacy, which included disease response rates (DRRs) and disease control rates (DCRs). The main outcomes are DRRs and DCRs as assessed by Response Evaluation Criteria in Solid Tumors 1.1 (RECIST 1.1) or PET Response Evaluation Criteria in Solid Tumors 1.0 (PERCIST 1.0). DRRs were assessed by the proportion of complete response (CR) + partial response (PR); DCRs were assessed by the proportion of complete response (CR) + partial response (PR) + stable disease (SD). Potential toxicity was collected according to the Common Terminology Criteria for Adverse Events 5.0 (CTCAE5.0).

### Statistical analysis

Stata16.0 was used for this meta-analysis. Generated forest plots were used for the analysis of DRRs and DCRs. *I* ^2^ statistic was used for the heterogeneity test. If there was no significant heterogeneity among studies (*I* ^2^ ≤ 50%, *p* < 0.10), a fixed-effects model was used to merge data. If there was significant heterogeneity among the studies (*I* ^2^ > 50%, *p* ≥ 0.10), the random-effects model was used to merge the data. In addition, subgroup analyses were carried out to explore the efficacy of patients who had previously received ^177^Lu PRRT. The funnel plot and Egger’s test were used to evaluate the publication bias of the studies, and *p* < 0.05 was considered statistically significant.

## Results

### Literature search

**Figure 1:**
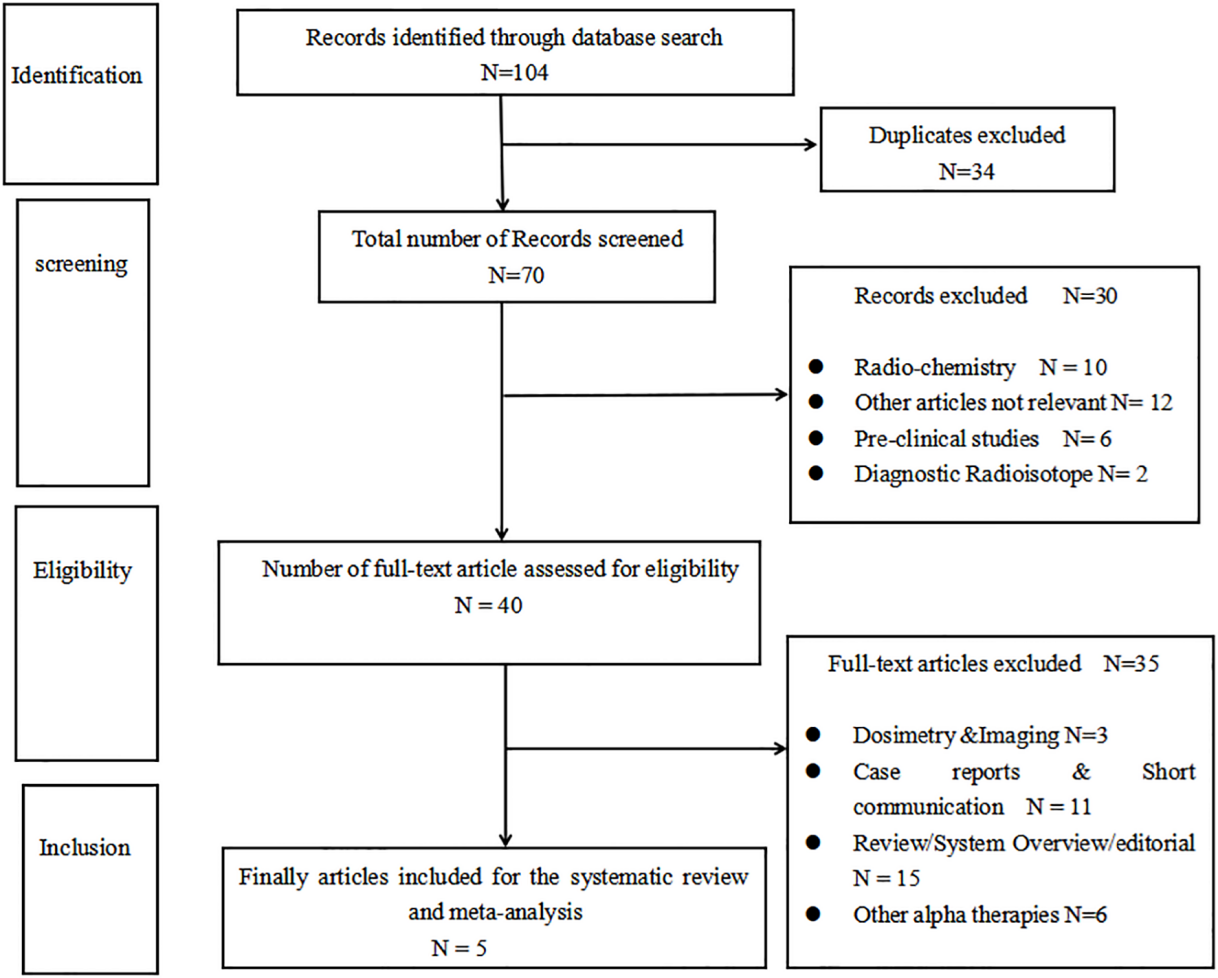
Flowchart of literature screening.

According to the search strategy, a total of 104 records were identified. Thirty-four duplicate records were excluded, and 30 articles were excluded by reading the title and the abstract. By further reading the full articles, five articles (17–21) that met the inclusion criteria were included. There is a flowchart that details how the articles were selected in **Figure 1**.

### Quality assessment

Based on the JBI Critical Appraisal Checklist for Case Series, five clinical studies were assessed, comprising 10 items. In Ballal et al., the case series that have consecutive inclusion of participants were not clear. The demographic information from the presenting sites/clinics was not clearly reported in all studies. The assessment results are provided in **Table 1**.

**Table 1:** Quality assessment of the included studies based on the JBI critical appraisal checklist for case series.

Study	Q1	Q2	Q3	Q4	Q5	Q6	Q7	Q8	Q9	Q10	Final decision
Yadav MP et al., 2021 (17)	Y	Y	Y	Y	Y	Y	Y	Y	U	Y	include
Ballal S et al., 2019 (18)	Y	Y	Y	Y	Y	Y	Y	Y	U	Y	include
Yang H et al., 2024 (19)	Y	Y	Y	Y	Y	Y	Y	Y	U	Y	include
Ballal S et al., 2022 (20)	Y	Y	Y	U	Y	Y	Y	Y	U	Y	include
Demirci E et al., 2023 (21)	Y	Y	Y	Y	Y	Y	Y	Y	U	Y	include

Q1–Q10: Q1, Were there clear criteria for inclusion in the case series? Q2, Was the condition measured in a standard, reliable way for all participants included in the case series? Q3, Were valid methods used for the identification of the condition for all participants included in the case series? Q4, Did the case series have consecutive inclusion of participants? Q5, Did the case series have a complete inclusion of participants? Q6, Was there clear reporting of the demographics of the participants in the study? Q7, Was there clear reporting of clinical information of the participants? Q8, Were the outcomes or follow-up results of cases clearly reported? Q9, Was there clear reporting of the presenting site(s)/clinic(s) demographic information? Q10, Was statistical analysis appropriate?

### Study characteristics

A total of five studies consisting of 153 patients were included in the analysis.

The total treatment cycles ranged from 1 to 9, and the follow-up time was from 5 to 41 months. Four studies have previously reported the use of ^177^Lu-PRRT in patients. RECIST 1.1 criteria were used to evaluate therapeutic efficacy in four studies (17, 18, 20, 21), and PERCIST 1.0 was used in one study (19). Toxicity was reported in four studies. In two studies (17, 20), capecitabine was used as a radiosensitizer, and amino acids were used to protect the kidneys in all studies, as shown in **Tables 2** and **3**.

**Table 2:** Characteristics of studies included in this meta-analysis.

Study and year	Study design	Response criteria	Patients (M/W)	Primary tumor type	Ki67	Prior therapies	Distant metastasis
Yadav MP et al., 2021	P	RECIST 1.1	9 (6/3)	Paraganglioma: 9	<3%: 3 3–20%: 3 >20%: 1 NA: 2	Surgery: 6 Chemotherapy: 1 Radiotherapy: 5 ^131^I-MIBG therapy: 2 ^177^Lu-PRRT: 7	Lymph node: 8 Bone: 6 Lung: 3 Liver: 2 Duodenum: 1
Ballal S et al., 2019	P	RECIST 1.1	32 (15/17)	Pancreatic NET: 16 Foregut NET: 7 Midgut NET: 3 Hindgut NET: 1 Unknown primary: 5	<3%: 11 3%–20%: 16 >20%: 3 NA: 2	Surgery: 10 Sandostatin (LAR/short-acting):28 Chemotherapy: 12	Lymph node: 24 Bone: 12 Liver: 29 Duodenum: 1
Yang H et al., 2024 (19)	P	PERCIST 1.0	10 (7/3)	Adrenal glands pheochromocytoma: 3 Medullary thyroid carcinoma: 1 Pancreatic NET: 1 Tonsillar NET: 1 Paraganglioma: 4 Lung carcinoid: 1	<3%: 1 3%–20%: 8 >20%: 0 NA: 1	Surgery: 6 Chemotherapy: 5 Radiotherapy: 1 Endocrinotherapy: 1 Immunotherapy: 1 Targeted therapy: 6 ^177^Lu-PRRT: 6	Lymph node: 9 Bone: 6 Liver: 4 Lung: 4 Adrenal gland: 2 Muscle: 1 Subcutaneous: 1
Ballal S et al., 2022 (20)	P	RECIST 1.1	91 (54/37)	Pancreatic NET: 30 Gastric NET: 7 Appendiceal NET: 1 Ileal NET: 12 Duodenal NET: 13 Jejunal NET: 2 Colonic NET: 2 Rectal NET: 8 Abdominal NET with unknown primary: 16	<3%: 33 3%–20%: 48 >20%: 7 NA: 3	Surgery:20 Chemotherapy: 20 ^177^Lu-DOTATATE therapy: 57	Lymph node: 66 Bone: 25 Liver: 88
Demirci E et al., 2023 (21)	R	RECIST 1.1	11 (8/3)	Pancreatic NET: 3 Pulmonary NET: 1 Non-pancreatic gastroenteropancreatic NET: 3 Unknown primary tumor: 3 Paraganglioma: 1	<3%:2 3%–20%: 7 >20%: 0 NA: 2	Long-acting somatostatin analogs: 10 Chemotherapy: 11 Radioembolization/chemoembolization to liver: 6 MIBG treatment: 2 ^177^Lu-DOTATATE: 10	Lymph node: 8 Bone: 8 Liver: 10 Lung: 4

P, prospective; R, retrospective; NET, neuroendocrine tumor.

**Table 3:** The treatment characteristics of the included studies.

Study and year	Dose per cycle (kBq/kg)	Total cycles (median, range)	Treatment interval time	Follow-up time (median, range)	Cumulative activity (MBq) (median, range)
Yadav MP et al., 2021 (17)	100	3 (2-9)	8 week	22.5months(18–28 months)	42.4 ± 27 (15.54-86.6)
Ballal S et al., 2019 (18)	100	3 (1-5)	8 week	8 months (2–13 months)	22.550 ± 9.842 (7.770-44.400)
Yang H et al., 2024 (19)	100	3 (2-6)	8 week	14 months (7–22 months)	22.9 ± 9.5 (14.8-44.4)
Ballal S et al., 2022 (20)	100–120	4(1-10)	8 week	24months(5-41month)	35.52 (21.64-59.47)
Demirci E et al., 2023 (21)	100–120	1(1-3)	NA	NA	8.2 ± 0.6 (7.5-10.0)

### Therapeutic efficacy

**Figure 2:**
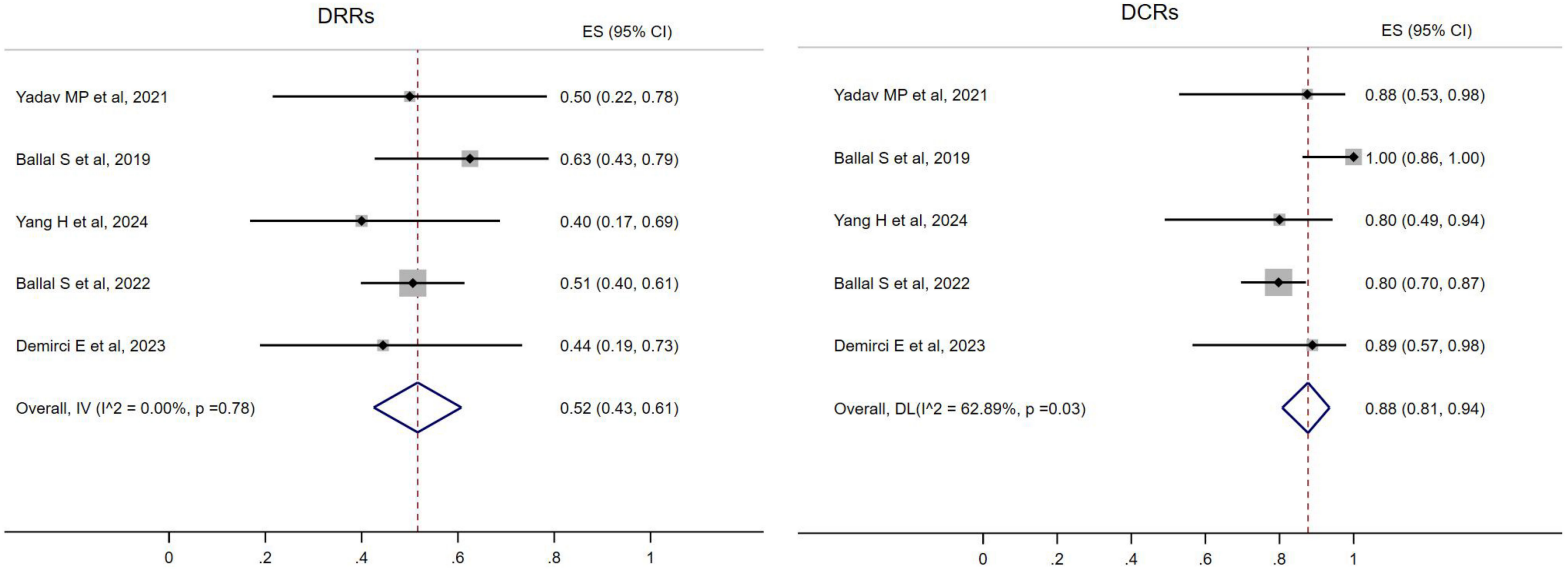
Forest plot of the proportions of disease response rates (DRRs) and disease control rates (DCRs) for all.

All five studies reported the treatment response of DRRs and DCRs. A fixed-effects model (*I* ^2^ = 0.00%, *p* = 0.78) was used and the pooled proportion of DRRs was 0.52 (95% CI, 0.43–0.61). A random-effects model (*I* ^2^ = 62.89%, *p* = 0.03) was used and the pooled proportion of DCRs was 0.88 (95% CI, 0.81–0.94), as shown in **Figure 2**.

**Figure 3:**
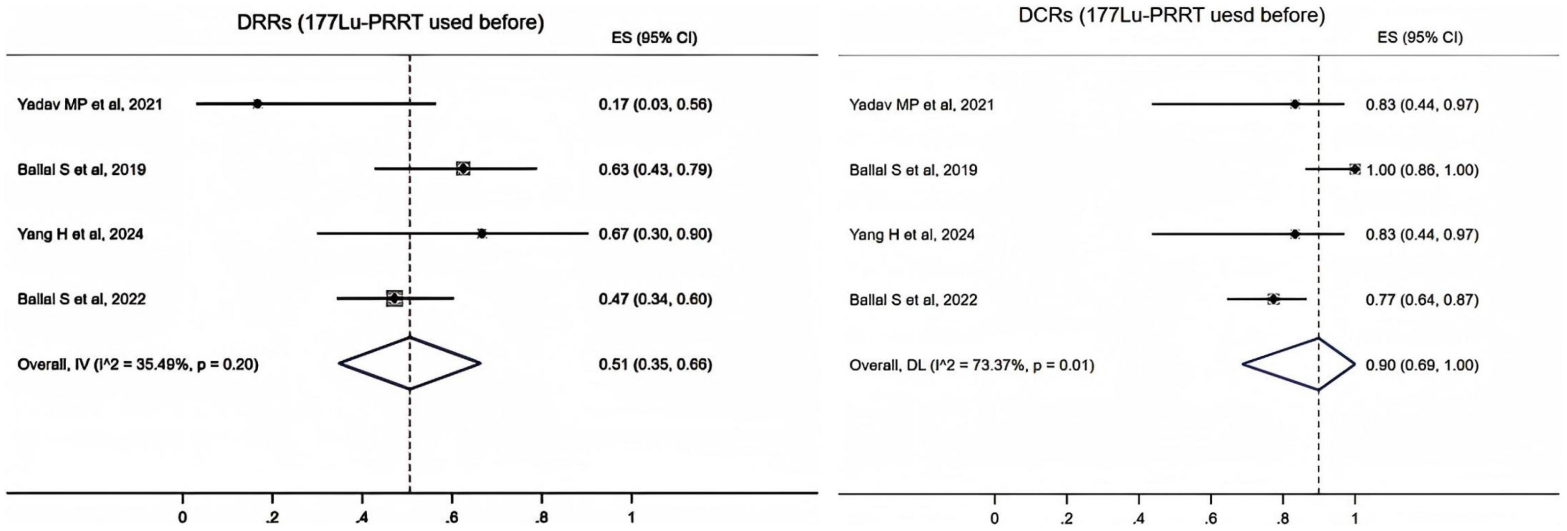
Forest plot of the proportions of disease response rates (DRRs) and disease control rates (DCRs) for patients with ^177^Lu-PRRT.

In four studies (17–20), 89 patients reported the use of ^177^Lu-PRRT before ^225^Ac-DOTATATE. A fixed-effects model (*I* ^2^ = 35.49%, *p* = 0.2) was used and the pooled proportion of DRRs was 0.51 (95% CI, 0.35–0.66). A random-effects model (*I* ^2^ = 73.37%, *p* = 0.01) was used and the pooled proportion of DCRs was 0.9 (95% CI, 0.69–1.00) (**Figure 3**).

**Figure 4:**
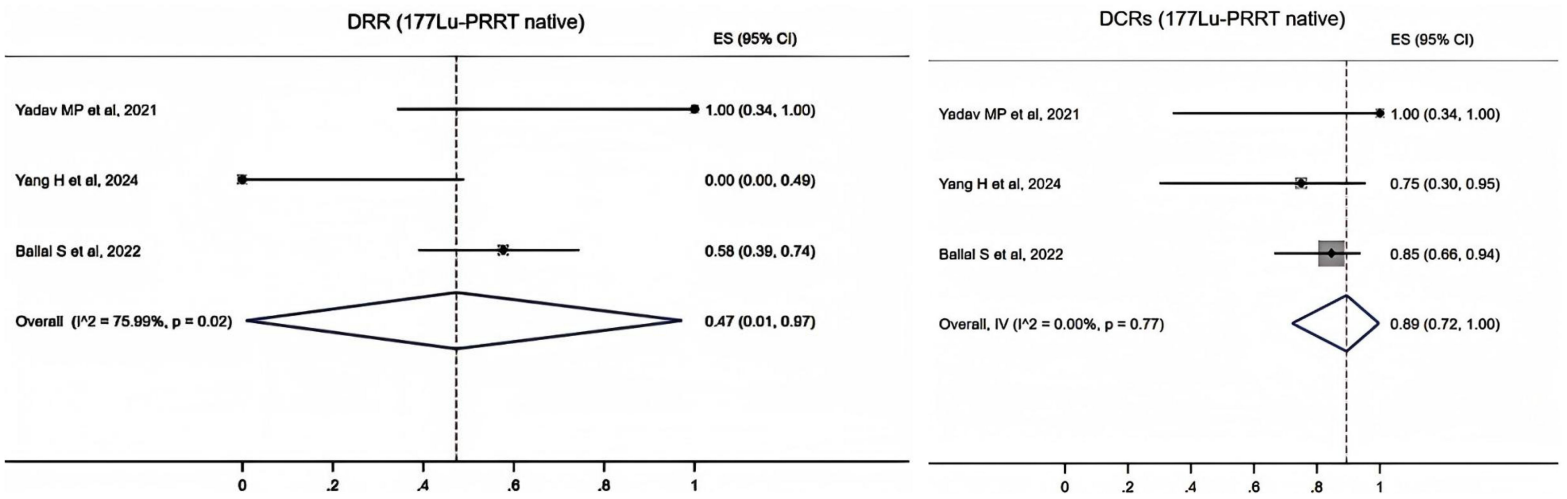
Forest plot of the proportions of disease response rates (DRRs) and disease control rates (DCRs) for patients with ^177^Lu-PRRT native.

The data of ^177^Lu-PRRT native patients were extracted from three studies. It showed that the pooled proportion of DRRs was 0.47 (95% CI, 0.01–0.97) using a random-effects model (*I* ^2^ = 75.99%, *p* = 0.02). The pooled proportion of DCRs was 0.89 (95% CI, 0.72–1.00) using a fixed-effects model (*I* ^2^ = 0.00%, *p* = 0.77) (**Figure 4**).

### Toxicity

**Figure 5:**
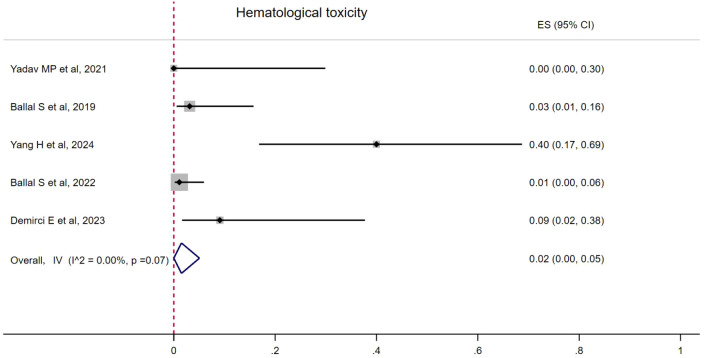
Forest plot of the proportions of hematological toxicity.

Hematological toxicity was seen in four studies, with seven patients (18–20). The pooled proportion of hematological toxicity was 0.02 (95% CI, 0.00–0.05) using a fixed-effects model (*I* ^2^ = 0.00%, *p* = 0.07). Nephrotoxicity was seen in two patients and no hepatotoxicity was reported (**Figure 5**). Toxicity details are summarized in **Table 4**.

**Table 4:** Treatment-related toxicity after treatment.

	Hematological toxicity			Nephrotoxicity	Hepatotoxicity
Study and year	Anemia	Leucopenia	Thrombocytopenia		
Yadav MP et al., 2021 (17)	/	/	/	/	/
Ballal S et al., 2019 (18)	/	Grade I: 1	/	Grade I: 1	/
Yang H et al., 2024 (19)	Grade I: 3	Grade I: 1	/	/	/
Ballal S et al., 2022 (20)	/	/	Grade III: 1	/	/
Demirci E et al., 2023 (21)	Grade II: 1			Grade II: 1	/

The pooled proportion therapeutic efficacy and toxicity results are summarized in **Table 5**.

**Table 5:** Pooled proportion therapeutic efficiency and toxicity.

Effects	No. of studies	Model	Pooled proportion (95% Cl)	*I* ^2^ (%)	*p*
DRRs	5	Fixed effects	0.52 (0.43–0.61)	0.00	0.78
DCRs	5	Random effects	0.88 (0.81–0.94)	62.89	0.03
DRRs (^177^Lu-PRRT)	4	Fixed effects	0.51 (0.35–0.66)	35.49	0.2
DCRs (^177^Lu-PRRT)	4	Random effects	0.9 (0.69–1.00)	73.37	0.01
DRRs (^177^Lu-PRRT native)	3	Random effects	0.47 (0.01–0.97)	75.99	0.02
DCRs (^177^Lu-PRRT native)	3	Fixed effects	0.89 (0.72–1.00)	0.00	0.77
Hematologic toxicity	5	Fixed effects	0.02 (0.00–0.05)	0.00	0.07

### Publication bias

**Figure 6:**
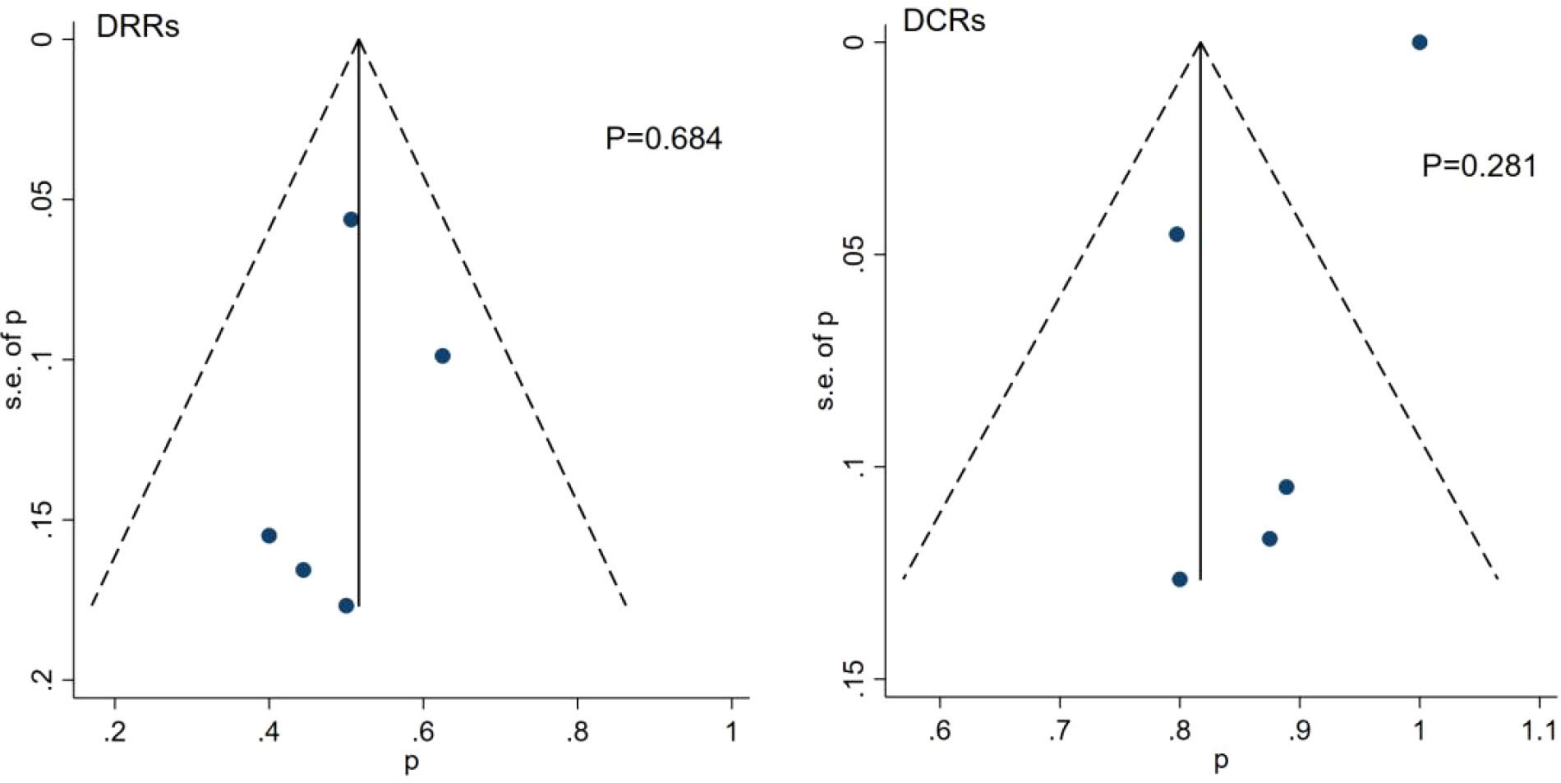
Funnel plot and Egger’s test for the publication bias of DRRs and DCRs.

Funnel plots and the Egger’s test were used to assess the publication bias of the studies. The results showed that there was no significant publication bias among these studies (**Figure 6**).

## Discussion

^225^Ac-DOTATATE has demonstrated immense potential in TAT for NETs in clinical practice. Our study included five research articles on the treatment of NETs with ^225^Ac-DOTATATE, with a focus on analyzing the therapeutic efficacy and toxicity of ^225^Ac-DOTATATE in advanced metastatic NETs. The tumor types included paraganglioma (PGL), gastroenteropancreatic neuroendocrine tumors (GEP-NETs), and adrenal gland pheochromocytoma, among others. Our meta-analysis showed that 52% of patients achieved DRRs, and 88% of patients exhibited DCRs following treatment with ^225^Ac-DOTATATE. In contrast, published meta-analyses on ^177^Lu-PRRT reported the DRRs ranging from approximately 20% to 35% (22, 23). Patients who had not previously undergone ^177^Lu-PRRT and directly received ^225^Ac treatment achieved a DRR of 47%, which is higher than 18% and 43% observed in the NETTER-1 trial and NETTER-2 trial for patients treated with ^177^Lu-DOTATATE (24, 25). Among the 89 patients who had previously undergone ^177^Lu-PRRT, they either opted for ^225^Ac due to disease progression after ^177^Lu treatment or discontinued ^177^Lu after reaching the maximum tolerated dose. The results showed that in these patients, the DRR was 51% and the DCR was 90% following ^225^Ac-DOTATATE therapy. ^177^Lu emits beta particles, which, despite their relatively wide range of action, have lower energy and may contribute to the development of resistance in tumor cells. Potential mechanisms for this resistance include the downregulation of receptor expression, enhanced DNA repair mechanisms in tumor cells, and changes in the tumor microenvironment (26). Because of their high LET (~100 keV/μm), alpha particles induce DNA double-strand breaks (DSBs) that are typically difficult for tumor cells to repair. Additionally, they exhibit strong cytotoxic effects even against resistant tumor cells in a low proliferative state (11, 27). Therefore, for patients who have developed resistance or shown no response to targeted beta therapy, ^225^Ac-DOTATATE has demonstrated significant potential in overcoming resistance to ^177^Lu-PRRT (28). Furthermore, we observed that although the DRRs and DCRs were slightly higher in patients who had undergone prior ^177^Lu-PRRT compared to ^177^Lu-naive patients, the results should be interpreted with caution due to the small sample size of the included studies.

The incidence of hematological toxicity was 2%, with grade I–II hematological toxicity observed in six patients, and grade III thrombocytopenia occurring in one patient.

Only grade I or grade II nephrotoxicity was observed in two patients (18, 21). Grade III/IV hematological or renal toxicity was not reported during the follow-up period, nor was any degree of hepatic toxicity observed. Kavanal et al. (29) reported a case of subclinical hypothyroidism following ^225^Ac-DOTATATE treatment in a patient with metastatic NETs, but no similar findings were noted in this study.

Four studies (17–20) have reported transient symptoms such as nausea, vomiting, and diarrhea during the treatment process due to amino acid infusion. However, these symptoms were resolved after the treatment was completed.

The average cumulative activity ranged from 7.5 to 86.6 MBq, with the longest follow-up period reaching 41 months. During the follow-up, patients exhibited good tolerance, and Grade III or higher adverse events were uncommon, transient, or unlikely to be related to the treatment. Further research is still needed to accurately measure the absorbed doses in target and non-target organs and to evaluate the maximum tolerated dose associated with alpha therapy. Four studies (17–20) demonstrated significant improvements in patients’ physical function, emotional state, and social functioning following treatment. As a salvage therapy, ^225^Ac-DOTATATE has shown remarkable potential in improving the quality of life and clinical symptoms of patients with NETs.

This meta-analysis has certain limitations. The sample sizes of the included studies were relatively small, and there were differences in the demographic characteristics of the patients. Because of limited data, it was not possible to explore the long-term prognostic efficacy of ^225^Ac-DOTATATE, such as overall survival (OS) and progression-free survival (PFS). This is a preliminary summary of ^225^Ac-DOTATATE in NETs. Owing to the limited number of participants included in the study, the conclusions drawn still lack robustness. Therefore, future high-quality, prospective, multicenter randomized controlled trials are needed to further clarify the optimal therapeutic dosage of ^225^Ac-DOTATATE and to explore combination treatment strategies in advanced metastatic NETs.

## Data Availability

The original contributions presented in the study are included in the article/supplementary material. Further inquiries can be directed to the corresponding author.
